# Metabolic reprogramming as a driver of immune escape in melanoma: implications for immunotherapy

**DOI:** 10.3389/fimmu.2026.1805144

**Published:** 2026-04-28

**Authors:** Hong Liang, Lina Han, Xiao Feng, Lian Zhang

**Affiliations:** 1 Department of Traditional Chinese Medicine Internal Medicine, Affiliated Hospital of Changchun University of Chinese Medicine, Changchun, China; 2 Department of Respiratory Medicine, Affiliated Hospital of Changchun University of Chinese Medicine, Changchun, China; 3 Department of General Surgery, Affiliated Hospital of Changchun University of Chinese Medicine, Changchun, China

**Keywords:** immune escape, immunotherapy resistance, melanoma, metabolism, tumor microenvironment

## Abstract

Melanoma has long served as a paradigm for cancer immunotherapy due to its high immunogenicity and the transformative impact of immune checkpoint blockade. However, durable clinical benefit remains confined to a subset of patients, with primary and acquired resistance remaining common. This plateau highlights a central unresolved question: how melanoma evades immune-mediated elimination despite reinvigorated antitumor immunity. Immune escape in melanoma cannot be fully explained by defects in antigen presentation, interferon signaling, or checkpoint regulation alone. Increasing evidence identifies tumor-intrinsic metabolic reprogramming as a dominant driver of immune dysfunction. By rewiring glucose, amino acid, lipid, and mitochondrial metabolism, melanoma cells create a metabolically restrictive microenvironment that suppresses effector T and NK cell function while favoring regulatory and myeloid immunosuppressive states through nutrient competition, inhibitory metabolite accumulation, and metabolite-driven signaling. In this Review, we synthesize recent advances establishing metabolic reprogramming as an organizing principle of immune escape in melanoma. We integrate how tumor metabolic programs shape immune cell fate, function, and spatial organization, and how metabolic crosstalk between tumor and immune compartments generates immune-resistant niches that persist despite checkpoint blockade. We further discuss emerging therapeutic strategies that target metabolic vulnerabilities, alone or in rational combination with immunotherapy, to overcome resistance by reconditioning the metabolic context of antitumor immunity. By reframing metabolism as a governing axis rather than a secondary hallmark of melanoma, this Review provides a conceptual and translational framework for the development of mechanism-guided immunotherapies with durable clinical impact.

## 1 Introduction

Melanoma represents one of the most immunogenic human malignancies and has served as a proving ground for modern cancer immunotherapy. The clinical introduction of immune checkpoint blockade, particularly antibodies targeting programmed cell death protein 1 (PD-1) and cytotoxic T-lymphocyte–associated antigen 4 (CTLA-4), has transformed the therapeutic landscape of advanced melanoma, yielding durable responses and unprecedented long-term survival in a subset of patients. Combination regimens have further improved response rates, establishing immune checkpoint inhibitors (ICIs) as the standard of care for unresectable and metastatic disease. Despite these advances, the majority of patients with melanoma either fail to respond to immunotherapy or eventually develop acquired resistance (1). In pivotal studies such as CheckMate 067, dual CTLA-4 and PD-1 blockade with nivolumab plus ipilimumab has yielded objective response rates around ~50%, underscoring both significant clinical benefit and a persistent need to overcome resistance mechanisms (2). These clinical realities underscore a central challenge in melanoma immunotherapy: how tumors evade immune-mediated destruction despite the presence of activated antitumor immune responses.

Tumor metabolism has emerged as a critical and previously underappreciated regulator of antitumor immunity (3). Melanoma cells undergo profound metabolic reprogramming to support rapid proliferation, survival under stress, and metastatic dissemination (4). However, these metabolic adaptations reshape the tumor microenvironment (TME) in ways that directly impair immune cell function, alter immune cell differentiation, and promote immunosuppressive niches (5). Together, these observations position melanoma as a model for understanding how intrinsic oncogenic and metabolic programs intersect with immune regulation. Melanoma is uniquely positioned at the intersection of oncogenic signaling, metabolic plasticity, and immune regulation (6). Oncogenic drivers frequently found in melanoma, including BRAF V600E (7) and NRAS mutations (8), not only promote tumor growth but also actively rewire glucose, amino acid, lipid, and mitochondrial metabolism. These metabolic programs influence cytokine production, antigen presentation, and immune checkpoint expression, thereby coupling intrinsic tumor biology to extrinsic immune evasion (9). Moreover, metabolic states within both tumor cells and immune cells critically determine responsiveness to immune checkpoint blockade. For example, high glycolytic flux and lactate accumulation have been associated with T cell dysfunction and reduced efficacy of PD-1–targeted therapies, while mitochondrial fitness of tumor-infiltrating lymphocytes (TILs) correlates with durable clinical responses (10, 11). Mechanistic studies increasingly show that metabolic reprogramming is a causal driver of immune escape. Spatial and temporal heterogeneity in metabolites, including lactate, kynurenine, and lipid derivatives, creates microenvironments that suppress effector immune function and establish immune-tolerant niches within melanoma lesions (12). In addition to metabolic reprogramming, melanoma exhibits remarkable phenotypic plasticity characterized by dedifferentiation and transcriptional rewiring. These processes, often driven by changes in MITF activity, AXL expression, and neural crest–like programs, are increasingly recognized as key contributors to resistance to immune checkpoint blockade. Importantly, emerging evidence suggests that these cell state transitions are tightly coupled to metabolic adaptations. Dedifferentiated melanoma cells frequently exhibit increased reliance on oxidative phosphorylation and fatty acid metabolism, while proliferative MITF-high states are associated with glycolytic metabolism. This bidirectional coupling between transcriptional states and metabolic programs provides a mechanistic bridge linking tumor plasticity to immune escape and therapeutic resistance.

Beyond revealing mechanistic insights, these findings underscore the translational potential of targeting tumor metabolism. Metabolic programs are inherently tunable and, in principle, amenable to pharmacologic intervention (13). Importantly, the complex and context-dependent nature of metabolic reprogramming suggests that simple, single-pathway approaches are unlikely to fully restore antitumor immunity. Instead, a deeper understanding of the spatial, temporal, and cell-type–specific dynamics of metabolic circuits is needed to rationally integrate metabolic modulation with immune-based therapies. Such approaches hold promise for reconditioning the tumor microenvironment and enhancing the effectiveness of immunotherapy, offering a conceptual framework for next-generation interventions.

In this Review, we synthesize recent advances elucidating how metabolic reprogramming in melanoma orchestrates immune escape. We first outline the major metabolic pathways rewired in melanoma, including glycolysis, amino acid metabolism, lipid metabolism, and mitochondrial oxidative phosphorylation. We then examine the mechanisms by which these metabolic alterations impair effector immune cells, promote immunosuppressive populations, and facilitate bidirectional metabolic crosstalk between tumor cells and the immune microenvironment. Finally, we discuss emerging therapeutic strategies that target metabolic vulnerabilities in melanoma, with a particular focus on combination approaches designed to enhance the efficacy and durability of immunotherapy. By integrating insights from tumor biology, immunology, and metabolism, this Review aims to highlight metabolism as a central axis of immune regulation in melanoma and a promising frontier for next-generation immunotherapeutic interventions.

## 2 Metabolic reprogramming in melanoma

### 2.1 Elevated glycolysis and lactate drive immune evasion in melanoma

**Figure 1:**
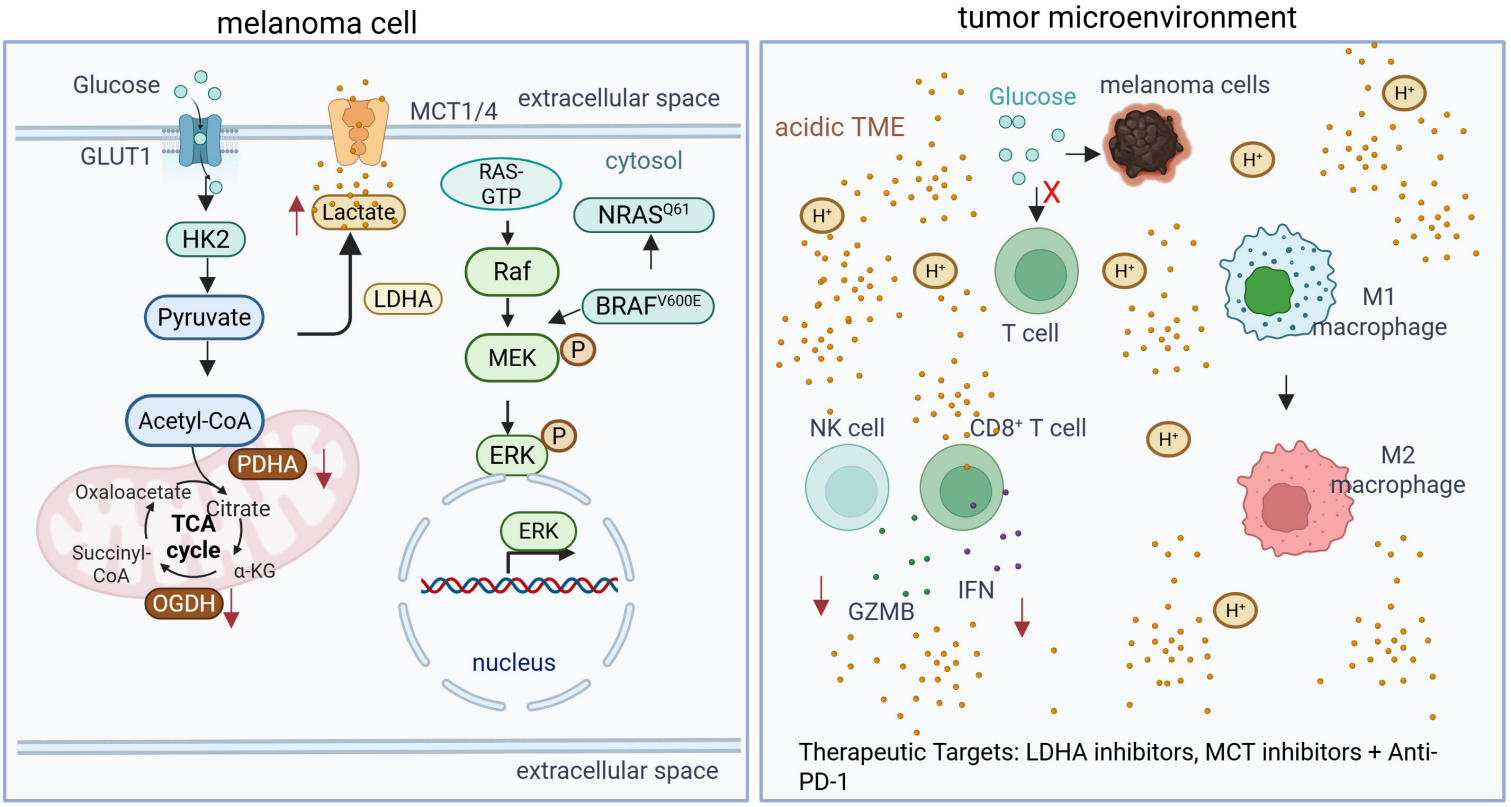
Schematic representation of metabolic cross-talk in the melanoma TME. Oncogenic signaling (e.g., BRAF V600E) shifts melanoma metabolism toward enhanced aerobic glycolysis, leading to glucose deprivation for infiltrating T cells and excessive extracellular lactate accumulation. This metabolic milieu suppresses effector T/NK cell functions and promotes immunosuppressive macrophage polarization through both metabolic competition and signaling mechanisms like histone lactylation.

Enhanced aerobic glycolysis is a hallmark of melanoma that drives immune evasion by both metabolic competition and lactate-mediated immunosuppression. Melanoma cells exhibit a pronounced Warburg phenotype, characterized by elevated glucose uptake and preferential conversion of pyruvate to lactate even under normoxic conditions (**Figure 1**). This state is reinforced by oncogenic signaling, notably BRAF V600E (14), which upregulates key glycolytic enzymes such as HK2, PFKFB3, and LDHA through MAPK-dependent transcriptional programs, as well as glucose transporters including GLUT1 (SLC2A1), enabling tumor cells to outcompete infiltrating immune cells for essential nutrients (15). NRASQ61-mutated melanomas depend on glucose metabolism for survival, with metabolic stress triggering a CRAF-to-BRAF signaling switch that activates PFKFB2/3 and PFK1, sustaining glycolytic flux; targeting this pathway with glycolysis inhibitors in combination with sorafenib effectively suppresses tumor growth in preclinical models (14). Mechanistically, inhibition of TCA cycle enzymes PDHA1 and OGDH shifts metabolism toward glycolysis and upregulates PD-L1 via AMPK–CREB–ATF3 signaling, generating a metabolically driven immunosuppressive environment; targeting this pathway enhances anti-PD-1 efficacy by coupling metabolic reprogramming to immune checkpoint modulation (16). Similarly, heightened glycolysis is linked to resistance to adoptive T cell therapy (ACT), as it impairs T cell–mediated tumor killing, whereas glycolysis inhibition restores antitumor immunity *in vitro* and *in vivo*, identifying glycolytic pathways as potential combinatorial therapeutic targets (17).

Tumor dependence on glycolysis further creates a metabolic advantage over infiltrating T cells, with high lactate dehydrogenase (LDH) expression driving lactate accumulation and limiting glucose availability in the TME (18). Pharmacologic LDH inhibition restores glucose accessibility to T cells, enhances effector function, suppresses immunoregulatory populations, and synergizes with immune checkpoint blockade to improve antitumor immunity in preclinical models. Mitochondrial DNA mutations in melanoma similarly promote a Warburg-like metabolic shift, reshaping the TME and enhancing immune infiltration, thereby sensitizing tumors to checkpoint blockade in a neutrophil-dependent manner (19).

A central consequence of elevated glycolysis is the accumulation of extracellular lactate, exported primarily via monocarboxylate transporters MCT1 and MCT4 (20). Lactate-mediated acidification of the TME directly suppresses CD8^+^ T and NK cell effector functions by inhibiting TCR signaling, reducing IFNG and GZMB expression, and impairing NFAT nuclear translocation (21). Lactate also disrupts glycolytic flux in activated T cells, limiting proliferation and cytokine production. CD8^+^ T cells in the TME adapt by upregulating MCT11, increasing lactate uptake and promoting exhaustion; targeting MCT11 genetically or pharmacologically restores T cell cytotoxicity and suppresses tumor growth, highlighting a direct metabolic mechanism of immune suppression (22).

Beyond nutrient competition, lactate functions as a signaling molecule. Uptake via MCT1 in T cells and dendritic cells induces histone lactylation, reprogramming gene expression toward immune tolerance (23). In melanoma-associated macrophages, lactate stabilizes HIF-1α and induces ARG1 and VEGF, promoting an M2-like immunosuppressive phenotype and reinforcing immune exclusion (24). Clinically, elevated tumor lactate correlates with poor responses to PD-1 blockade, underscoring glycolytic reprogramming as a metabolic barrier to effective immunotherapy (25).

### 2.2 Amino acid metabolism

**Figure 2:**
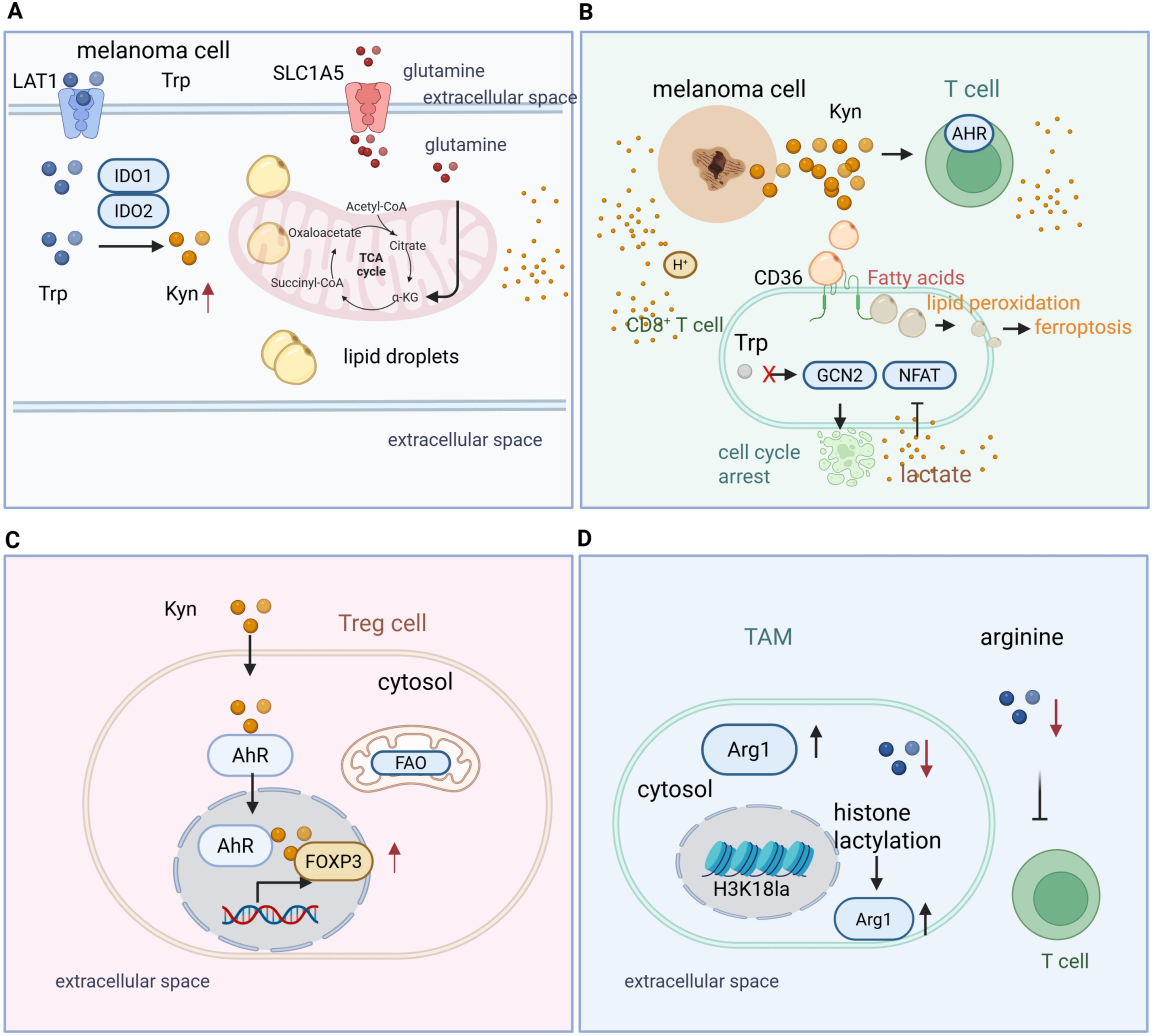
Metabolic reprogramming of amino acids and lipids orchestrates an immunosuppressive landscape in the melanoma TME. Melanoma cells and various immune subsets undergo profound metabolic adaptations that collectively impair antitumor immunity. **(A)** Melanoma cell intrinsic amino acid and lipid metabolism. Oncogenic signaling drives the upregulation of IDO1/2 and TDO2, catalyzing the conversion of Tryptophan (Trp) to Kynurenine (Kyn). Elevated lipid synthesis and uptake via CD36 promote lipid droplet accumulation, providing substrates for membrane biogenesis and pro-tumorigenic signaling. **(B)** Metabolic constraint of CD8^+^ T cells. Depletion of essential amino acids (Trp, Arg) in the TME activates the GCN2 stress kinase pathway, leading to ribosomal stalling and cell cycle arrest. Simultaneously, CD36-mediated uptake of long-chain fatty acids induces lipid peroxidation and mitochondrial dysfunction, ultimately driving T cell exhaustion and ferroptosis. **(C)** Metabolic fitness of Regulatory T cells (Tregs). Kynurenine acts as a signaling ligand for the Aryl Hydrocarbon Receptor (AhR), which translocates to the nucleus to drive *FOXP3* expression. Unlike effector T cells, Tregs utilize fatty acid oxidation (FAO) to maintain suppressive functions in nutrient-depleted environments. **(D)** Macrophage polarization and epigenetic rewiring. High lactate levels in the TME induce histone lactylation (e.g., H3K18la), promoting the transcription of M2-associated genes such as *ARG1* and *VEGF.* Arginase 1 (ARG1) further depletes extracellular arginine, reinforcing a self-perpetuating cycle of immune exclusion and suppression.

Melanoma-driven alterations in amino acid metabolism suppress antitumor immunity by depriving effector lymphocytes of essential nutrients while generating immunosuppressive metabolites (**Figure 2**).

#### 2.2.1 Tryptophan metabolism drives immune suppression and modulates antitumor T cell responses

Tryptophan metabolism, mediated by indoleamine 2,3-dioxygenase 1 (IDO1) and tryptophan 2,3-dioxygenase (TDO2), is a central mechanism of immune evasion in melanoma (26). Melanoma cells and tumor-associated antigen-presenting cells frequently upregulate IDO1 in response to inflammatory cues such as IFN-γ, generating a paradoxical negative feedback loop. Depletion of tryptophan in the tumor microenvironment activates the GCN2 kinase pathway in T cells, leading to cell cycle arrest and anergy, while accumulation of the catabolite kynurenine activates the aryl hydrocarbon receptor (AHR) in both T cells and regulatory T cells (Tregs), skewing differentiation toward an immunosuppressive phenotype and promoting FOXP3 expression (26, 27). Tryptophan deprivation also induces AHR overexpression and enhances GCN2/LAT1-mediated kynurenine uptake, further potentiating Treg differentiation and reinforcing immune suppression (27). High melanoma tryptophan metabolism, driven by enzymes such as TPH1 and the LAT1 transporter, deprives TILs of tryptophan and correlates with poor response to PD-1 blockade, highlighting amino acid competition as a mechanism of immunotherapy resistance (28). Notably, intratumoral Lactobacillus reuteri releases the tryptophan catabolite indole-3-aldehyde (I3A), which activates AhR in CD8^+^ T cells, enhances interferon-γ production, and potentiates immune checkpoint inhibitor efficacy, with dietary tryptophan further amplifying this microbial-host crosstalk (29).

Clinical studies and patient analyses reveal broad alterations across tryptophan metabolic pathways, including enhanced kynurenine and serotonin catabolism, which favor immunosuppressive metabolites and NAD^+^ production, underscoring tryptophan metabolism as a key mechanism of tumor immune evasion (30). While IDO1 catalysis of tryptophan to kynurenine creates an immunosuppressive microenvironment, early-phase trials combining IDO1 inhibitors with PD-1 blockade showed limited benefit; alternative targets such as TDO2 and the AHR pathway remain promising strategies to overcome tryptophan-mediated immune suppression (31).

Mechanistically, persistent IL-2 signaling drives CD8^+^ T cell exhaustion through STAT5 activation, inducing tryptophan hydroxylase 1 and promoting AhR nuclear translocation, thereby upregulating inhibitory receptors and suppressing effector functions (32). IFN-γ–induced IDO1 expression also depletes tryptophan, causing ribosomal stalling and frameshifting that generate aberrant peptides; these can prime T cells but simultaneously diversify the tumor peptidome and contribute to immune evasion (12). Similarly, tryptophan depletion induces ribosomal frameshifting (“sloppiness”) amplified by oncogenic MAPK signaling, producing aberrant peptides that can be presented to T cells and recognized for cytotoxic killing, linking amino acid stress to immune modulation (33).

#### 2.2.2 Glutamine metabolism suppresses antitumor immunity while promoting melanoma growth

Glutamine metabolism represents a central amino acid pathway that simultaneously sustains melanoma cell proliferation and constrains antitumor immune responses through metabolic competition and signaling crosstalk within the TME. Melanoma cells exhibit enhanced glutamine uptake and utilization, frequently mediated by upregulation of the glutamine transporter SLC1A5 and conversion of glutamine to glutamate by glutaminase (GLS), thereby fueling the tricarboxylic acid (TCA) cycle and supporting anabolic and bioenergetic demands. This preferential allocation of glutamine to tumor cells creates a nutrient-depleted TME, in which infiltrating CD8^+^ T cells are deprived of a critical substrate required for mTORC1 signaling, proliferation, and effector differentiation, establishing a metabolic “tug of war” that undermines antitumor immunity.

At the tumor cell–intrinsic level, multiple oncogenic and metabolic programs converge to reinforce glutamine dependence in melanoma. Activating VEGFR2^R1051Q mutations enhance glutamine uptake and metabolism, increase ATP production, and impose a state of glutamine addiction that represents a potential metabolic vulnerability (34). In BRAF-mutant tumors, STAG2 inactivation reprograms glutamine metabolism by destabilizing c-Myc through ERK/AKT/GSK3β signaling, leading to downregulation of glutamine transport and utilization pathways and increased sensitivity to glutamine deprivation or glutaminase inhibition (35). In addition, TFEB functions as a master metabolic regulator in melanoma by coupling ERK signaling with coordinated control of glucose, glutamine, and cholesterol metabolism; TFEB inhibition suppresses ERK1/2 activity via DUSP1 induction, blocks CDK4-driven proliferation, and reduces glutamine uptake and glutaminolysis, leading to metabolic shutdown and tumor growth arrest (36). In parallel, moderate ROS levels activate mTOR signaling, thereby enhancing glutamine metabolism and promoting melanoma cell proliferation and survival, highlighting a ROS–mTOR–glutamine axis that supports tumor growth (37). Consistent with these mechanistic findings, transcriptomic analyses have identified glutamine metabolism–related gene signatures that stratify melanoma metabolic states and correlate with immune cell infiltration, immune checkpoint expression, and clinical prognosis, linking glutamine utilization to both tumor progression and immune suppression (38).

Beyond direct glutamine addiction, melanoma cells flexibly engage alternative nutrient pathways that indirectly reshape glutamine dependency and immune pressure. ACSS1-dependent acetate utilization fuels mitochondrial metabolism while reducing reliance on glucose and glutamine, thereby sustaining melanoma growth and metastasis and reinforcing tumor metabolic dominance within the TME (39). These adaptive metabolic programs collectively enhance tumor fitness while exacerbating nutrient competition with immune cells.

Glutamine metabolism in melanoma is further shaped by metabolic crosstalk with stromal components of the TME, particularly cancer-associated fibroblasts (CAFs). Tumor-instructed glutamine synthesis by CAFs promotes polarization of pro-tumorigenic TAMs, establishing a metabolic axis that supports tumor growth and suppresses antitumor immunity (40). Consistently, CAFs sustain melanoma survival under glutamine limitation by engaging in glutamine metabolic coupling with tumor cells. Targeted disruption of glutamine uptake and synthesis in both CAFs and melanoma cells breaks this metabolic symbiosis, remodels the extracellular matrix, and suppresses tumor progression (41). Together, these findings position CAF-driven glutamine metabolism as a key amplifier of immunosuppressive TME architecture.

Importantly, glutamine metabolism in melanoma exhibits strong context dependency, extending beyond a simple pro-tumor paradigm. In contrast to glutamine deprivation–induced dedifferentiation, dietary glutamine supplementation increases intratumoral α-ketoglutarate levels, drives epigenetic reprogramming through reduced H3K4me3 methylation, suppresses oncogenic transcriptional programs, and enhances sensitivity to BRAF inhibition, ultimately restraining melanoma growth (42). These observations highlight glutamine availability as a regulator of melanoma cell state and therapeutic responsiveness.

#### 2.2.3 Arginine metabolism drives immune suppression and metabolic constraint in melanoma

Arginine metabolism constitutes a central metabolic axis through which tumor-associated myeloid populations suppress antitumor immunity (43). In TME, high activity of arginine-catabolizing enzymes such as arginase 1 (ARG1) and inducible nitric oxide synthase (iNOS/NOS2), predominantly expressed by myeloid-derived suppressor cells (MDSCs) and TAMs, depletes extracellular arginine—a semi-essential amino acid essential for T cell proliferation and effector differentiation. Arginine deprivation imposes a metabolic blockade on activated T cells by limiting protein synthesis and mTORC1 signaling, leading to cell cycle arrest in G0–G1 phase, downregulation of TCR ζ chain (CD3ζ), and impaired T cell activation (44). Concurrently, iNOS-mediated conversion of arginine to nitric oxide (NO) and citrulline exerts nitrosative stress on T cells and modulates dendritic cell function, amplifying immunosuppression (45). Experimental evidence underscores the impact of arginine metabolism on T cell immunity. Elevated ARG1 activity correlates with reduced CD8^+^ T cell proliferation and diminished IFNγ production, while arginine supplementation restores metabolic fitness, promotes mitochondrial respiration, and supports long-lived central memory–like T cells with enhanced antitumor activity (46). MDSCs also dynamically increase arginine uptake and catabolism in response to cytokines such as IFNγ, IL-4, and IL-10, further reducing extracellular arginine availability and reinforcing immunosuppression.

Therapeutic strategies exploiting arginine metabolism vulnerabilities have shown promise. ADI-PEG20–mediated arginine deprivation selectively targets arginine-auxotrophic melanoma cells, and its combination with TRAIL enhances apoptosis by coupling metabolic stress with death receptor signaling (47). Fusion of microbial-derived ADI with the cell-penetrating protein 30Kc19α improves enzyme stability, solubility, and intracellular delivery, overcoming resistance from re-expression of argininosuccinate synthase 1 (ASS1) and promoting tumor cell apoptosis (48). In BRAF inhibitor–resistant melanoma, RNF44-mediated degradation of AMPK-α1 shifts tumor metabolism from glucose to arginine dependence, sensitizing cells to arginine deprivation and revealing a potential therapeutic vulnerability (49).

Epigenetic regulation of arginine-related pathways further shapes antitumor immunity. PRMT5 suppresses cGAS/STING signaling and NLRC5-dependent MHC class I expression, limiting interferon and chemokine production; inhibition of PRMT5 restores immune signaling and synergizes with immune checkpoint blockade (ICB) (50). Similarly, PRMT1 inhibition or deletion activates endogenous retroviral element–derived double-stranded RNA, triggering interferon responses, increasing CD8^+^ T cell infiltration, and enhancing PD-1 blockade efficacy (51). PRMT7 ablation elevates interferon signaling, antigen presentation, and ERV expression, sensitizing melanoma to ICB (52). SHARPIN enhances PRMT5 activity through the SKI/SOX10 axis, promoting tumor growth and highlighting PRMT5 as a key immunometabolic node (53). Beyond methylation, arginine post-translational modifications contribute to metabolic rewiring. PADI1 and PADI3 mediate citrullination of PKM2, reprogramming glycolysis to sustain proliferation under metabolic stress (54). Additionally, Rbfox3 drives the formation of MDSC-like tumor cells that produce arginine, NO, and ROS, generating an immunosuppressive microenvironment and suppressing T and NK cell activity, while targeting Rbfox3 enhances ICB efficacy (55).

Collectively, these findings demonstrate that arginine metabolism not only directly constrains T cell effector function but also orchestrates broader immunosuppressive networks through myeloid cells, epigenetic regulators, and post-translational modifications. Therapeutic targeting of arginine catabolism and associated pathways holds potential to restore T cell–mediated antitumor immunity and improve responses to immunotherapy in melanoma.

### 2.3 Lipid metabolism reprogramming suppresses antitumor immunity in melanoma

#### 2.3.1 Melanoma lipid metabolic reprogramming

Melanoma cells rewire lipid metabolism to support proliferation, survival, and therapy resistance, relying on both enhanced endogenous lipid synthesis and exogenous lipid uptake. Upregulation of fatty acid transporters such as CD36, lipogenic transcription factors SREBP1 and SREBP2, and enzymes involved in fatty acid and cholesterol biosynthesis enables melanoma cells to sustain membrane biogenesis, β-oxidation, and energy production, particularly under nutrient-limited conditions, creating a lipid-rich TME that fuels tumor growth and metastasis (56). TNFα promotes cutaneous melanoma progression by activating the PLEKHA5-FCRLA axis, enhancing neutral lipid storage and energy metabolism. PLEKHA5 upregulation increases cholesterol ester and triglyceride levels while reducing ceramide and sphingosine, linking altered lipid metabolism to malignant behavior (57).

Melanoma cells also exploit peroxisomes and UDP-glucose ceramide glucosyltransferase (UGCG) to survive MAPK inhibitor therapy, and disruption of these pathways selectively eliminates drug-tolerant CD36^+^ melanoma cells, sensitizing tumors to MAPK inhibition (58). Dysregulated lipid composition in BRAF inhibitor–resistant cells, including reduced saturated fatty acids and cholesteryl esters, further supports survival, while targeting key lipid metabolic enzymes such as ACAT2 or SOAT restores drug sensitivity and promotes ferroptosis (59).

In addition, melanoma cells utilize stromal and hepatocyte-derived lipids to sustain growth. Fructose metabolism in hepatocytes generates lysophosphatidylcholines (LPCs) that are taken up by tumor cells to support phosphatidylcholine synthesis and membrane biogenesis, thereby indirectly promoting proliferation (60, 61). Autophagy-mediated lipid droplet degradation (lipophagy) in lymphatic endothelial cells supports mitochondrial fatty acid oxidation, maintains acetyl-CoA levels, and PROX1-driven lymphatic gene expression; impaired lipophagy leads to lipid droplet accumulation, reduced mitochondrial metabolism, and defective lymphangiogenesis (62), illustrating how lipid metabolism in the microenvironment can influence tumor progression. Lipid droplets also serve as metabolic reservoirs in melanocytic melanoma cells, supporting proliferation through fatty acid oxidation and creating a metabolic vulnerability that can be therapeutically exploited (63).

#### 2.3.2 Lipid metabolism suppresses antitumor immunity

Reprogrammed lipid metabolism in melanoma establishes a metabolically hostile TME that impairs multiple layers of antitumor immunity. In CD8^+^ T cells, excessive uptake of long-chain fatty acids and oxidized lipids via CD36 induces intracellular lipid accumulation, promotes lipid peroxidation, and triggers ferroptotic or ferroptosis-like dysfunction. This reduces effector cytokine production (IFN-γ, TNF-α) and upregulates inhibitory receptors including PD-1 and TIM-3, culminating in T cell exhaustion. Pharmacological blockade of CD36 or inhibition of lipid peroxidation restores T cell function and enhances responses to immune checkpoint blockade (64, 65). In addition, dysregulated lipid metabolism within T cells can drive effector T cell senescence. Inhibiting group IVA phospholipase A2 (PLA2G4A) reprograms T cell lipid metabolism, prevents senescence, and enhances immunotherapy efficacy in melanoma models (66), highlighting lipid metabolic pathways in T cells as actionable targets to boost antitumor immunity).

Beyond T cells, lipid accumulation compromises dendritic cell (DC) functionality, further dampening adaptive immunity. Tumor-associated DCs accumulate lipids that disrupt antigen processing, MHC class I/II trafficking, and costimulatory molecule expression (CD40, CD80, CD86), skewing T cell priming toward tolerance rather than activation. Dysregulated cholesterol metabolism and its oxidized derivatives (oxysterols) engage nuclear receptors such as LXRα in DCs, promoting recruitment of immunosuppressive myeloid populations while impairing DC migration and CD8^+^ T cell mitochondrial fitness, which exacerbates T cell exhaustion and limits PD-1/PD-L1 blockade efficacy (67).

Immunosuppressive cells in the TME exploit lipid metabolic programs to reinforce suppression. Regulatory T cells (Tregs) and M2-like TAMs preferentially utilize fatty acid oxidation (FAO) and activate CPT1A and PPARγ signaling to support survival and suppressive functions in the lipid-rich tumor microenvironment. Enhanced FAO in Tregs fuels oxidative phosphorylation and supports Foxp3 expression and immunosuppressive cytokine production such as IL-10 and TGF-β, promoting immune tolerance and inhibiting effector T cell recruitment. Activation of PPARγ has been shown to upregulate CD36 and CPT1-mediated fatty acid uptake and oxidation in Tregs, thereby stabilizing their suppressive phenotype and reinforcing their function in tumors. Parallel mechanisms operate in TAMs, where lipid metabolic reprogramming and PPAR signaling contribute to alternative (M2-like) polarization and immunosuppressive activity. Targeting lipid metabolic pathways such as FAO and PPARγ can destabilize Treg and TAM suppressive states and enhance antitumor immunity, highlighting lipid metabolism as a promising therapeutic target in cancer immunotherapy (68).

Finally, tumor-derived extracellular vesicles (EVs) amplify lipid-mediated immune suppression. EVs carrying PD-L1 promote T cell senescence by enhancing lipid droplet formation and cholesterol accumulation, thereby limiting the efficacy of adoptive T cell therapy and PD-L1 blockade (69). Collectively, these mechanisms illustrate how tumor-intrinsic and microenvironmental lipid metabolic rewiring converges to suppress antitumor immunity across multiple immune cell compartments, establishing a TME that favors melanoma progression.

#### 2.3.3 Lipid-dependent ferroptosis as a therapeutic vulnerability

Lipid metabolism intersects with ferroptosis, an iron-dependent, lipid peroxidation-driven form of regulated cell death, in a context-dependent manner that shapes therapeutic responses. In melanoma cells, dysregulated lipid metabolism modulates the expression of ferroptosis-associated genes including ACSL4, ALOX5, PTGS2, and LPCAT3, sensitizing cells to IFN-γ–induced lipid peroxidation and ferroptosis, thereby enhancing antitumor immunity and anti-PD-1 therapy efficacy (70). Conversely, excessive lipid peroxidation in CD8^+^ T cells induces ferroptotic dysfunction, highlighting the dual role of lipid peroxidation in tumor versus immune compartments.

Pharmacologic interventions targeting lipid metabolism can leverage this vulnerability. Mefloquine (Mef) upregulates LPCAT3 via IFN-γ–STAT1–IRF1 signaling, promoting lipid peroxidation–dependent ferroptosis in tumor cells and sensitizing melanoma and lung cancer to anti-PD-1 immunotherapy (70). Additionally, SREBP2-mediated induction of Transferrin in circulating melanoma cells reduces intracellular iron and reactive oxygen species, suppressing ferroptosis and promoting metastatic potential (56). These mechanistic insights reveal lipid metabolic nodes as actionable targets to potentiate ferroptosis and enhance immunotherapy efficacy.

### 2.4 Mitochondrial metabolism reprogramming undermines antitumor immunity in melanoma

#### 2.4.1 Mitochondrial plasticity supports tumor survival and therapy resistance

Melanoma cells exploit mitochondrial metabolic plasticity to adapt to therapeutic and nutrient stress. While traditionally considered glycolytic, subsets of melanoma cells rely on oxidative phosphorylation (OXPHOS) to maintain energy homeostasis, biosynthesis, and survival, especially under MAPK inhibitor therapy or immune checkpoint blockade resistance (71). Stromal-to-tumor mitochondrial transfer further enhances OXPHOS capacity, highlighting intercellular metabolic crosstalk. Therapeutically, targeting mitochondrial biogenesis or respiratory complexes can reduce tumor growth and sensitize resistant melanoma cells, emphasizing the central role of mitochondria in tumor persistence.

#### 2.4.2 Tumor OXPHOS shapes a metabolically restrictive TME

High mitochondrial oxidative metabolism in tumor cells creates localized hypoxia and metabolic competition, limiting oxygen and nutrient availability for TILs (72). This environment promotes T cell exhaustion, impairs proliferation, and reduces responsiveness to PD-1/PD-L1 blockade (73). Tumor OXPHOS-driven hypoxia also affects other immune cells: dendritic cells show reduced antigen presentation, and macrophages adopt immunosuppressive phenotypes, collectively reinforcing immune evasion. These findings link tumor-intrinsic mitochondrial programs to a hostile TME that restricts antitumor immunity.

#### 2.4.3 TIL Mitochondrial dysfunction and redox Imbalance promote exhaustion

Effective antitumor T cells require functional mitochondria for sustained effector activity and memory formation. Chronic antigen exposure, metabolic competition, and hypoxia impair TIL mitochondrial function, resulting in depolarized membranes, reduced spare respiratory capacity, and excessive ROS accumulation, which drive upregulation of inhibitory receptors (PD-1, TIM-3) and exhaustion. Modulating mitochondrial fitness—through PGC-1α overexpression, AMPK activation, or NAD^+^ supplementation (NR/NAM)—restores OXPHOS, decreases ROS, and enhances effector cytokine production (IFN-γ, TNF-α), improving T cell function and antitumor efficacy (74).

#### 2.4.4 Targeting mitochondrial pathways to recondition the immune microenvironment

Mitochondrial metabolism also governs the function of other immune populations, including DCs and macrophages, whose OXPHOS status influences antigen presentation and immune polarization. Therapeutic strategies that enhance mitochondrial biogenesis in effector cells or modulate ROS/redox balance can synergize with checkpoint blockade to overcome immunosuppression and improve tumor control. Collectively, these data highlight mitochondrial metabolism as a central node linking tumor resistance, immune dysfunction, and therapeutic opportunity in melanoma.

Collectively, these metabolic programs do not operate in isolation but form an interconnected network that dynamically regulates immune escape. Rather than acting as parallel pathways, glycolysis, amino acid metabolism, lipid metabolism, and mitochondrial function converge to establish a metabolically coordinated immunosuppressive ecosystem. For example, lactate accumulation not only suppresses T cell function but also reinforces lipid metabolic reprogramming in macrophages, while amino acid depletion reshapes epigenetic and transcriptional states that further stabilize immune resistance. This systems-level integration suggests that metabolic reprogramming should be conceptualized as a higher-order regulatory layer governing immune escape, rather than a set of independent metabolic alterations.

## 3 Targeting tumor metabolic dependencies to improve immunotherapy efficacy in melanoma

Tumor metabolism represents a tractable axis for therapeutic intervention in melanoma, particularly when combined with immune checkpoint blockade or other immunotherapies. One promising strategy is targeting glycolytic metabolism: pharmacologic inhibition of LDH reduces tumor glucose uptake and lactate efflux, thereby increasing glucose availability for infiltrating T cells, improving effector function, and destabilizing suppressive Treg activity. In preclinical melanoma models, LDH inhibitors combined with PD-1 blockade enhanced effector T cell infiltration and activation and controlled tumor progression more effectively than checkpoint inhibition alone, highlighting a metabolic means to rebalance nutrient competition in TME and overcome immunosuppressive metabolic barriers (**Table 1**).

**Table 1:** Therapeutic strategies targeting metabolic immune escape in melanoma.

Metabolic target/pathway	Therapeutic modality	Representative approaches	Development stage	Reference
Glycolysis/lactate export	Small-molecule inhibitors	LDHA inhibitors (Oxamate, FX11, GF), MCT1/4 inhibitors (e.g., AZD3965)	Preclinical – early clinical	(18)
	Combinatorial regimens with ICBs	LDHIs + anti-PD-1/CTLA-4	Preclinical	(82)
	Nanomedicine metabolic rewiring	Lactate oxidase nanocapsules to reduce lactate	Preclinical	(75)
	Lactate transporter blockade	MCT1 inhibitors (e.g., BAY-8002) to restore DC metabolic function	Preclinical	(83)
	LDHA targeting	Small-molecule LDHA inhibition to reduce lactate-mediated immunosuppression	Preclinical	(10)
Tryptophan metabolism	Enzyme inhibitors	IDO1/TDO2 inhibitors (e.g., Epacadostat)	Clinical (early)	(76)
	Combinatorial with ICIs	IDO1i + anti-PD-1	Clinical (phase I/II)	(84)
Arginine metabolism/microbiome	Combination targeted + ICI therapy	Neoadjuvant cobimetinib + atezolizumab ± vemurafenib, followed by adjuvant atezolizumab	Phase II clinical trial	(85)
Fatty acid oxidation/lipid metabolism	Metabolic modulators	CPT1A inhibition, FAO inhibitors	Preclinical	(79)
	Lipid uptake modulation	CD36 inhibition	Preclinical	(68)
Mitochondrial OXPHOS/ETC	ETC modulators	Mitochondrial translation & respiratory modulation	Preclinical	(71)
	Mitochondrial metabolism reinforcement	Promote TIL OXPHOS to enhance function	Preclinical	(86)
Amino acid metabolism (except Trp)	Arginine/Arg1 targeting	Arg1 inhibitors/arginine depletion blockade	Preclinical	(87)
Immune cell metabolism/metabolic reprogramming	Cell engineering/metabolic programming	PGC1α/AMPK/mito fitness boosters in ACT cells	Preclinical	(74)
	Nutrient competition modulation	Glucose, amino acid restoration	Preclinical	(18)

In addition to inhibiting lactate production, therapeutic strategies targeting lactate transport are also being explored. Small-molecule inhibitors of monocarboxylate transporters (MCT1/4), such as AZD3965 and BAY-8002, can block lactate export from tumor cells, thereby reducing extracellular acidification and restoring dendritic cell and T-cell metabolic fitness within the tumor microenvironment (18). Emerging nanomedicine approaches, including lactate-oxidase–based nanocapsules that enzymatically deplete intratumoral lactate, have further demonstrated the feasibility of metabolically rewiring the tumor microenvironment to enhance antitumor immunity in preclinical models (75).

Targeting amino acid metabolism also shows therapeutic potential. Among these pathways, tryptophan metabolism has received considerable attention due to the immunosuppressive effects of the kynurenine–AHR axis. Pharmacologic inhibition of enzymes such as IDO1 or TDO2 has been explored to prevent tryptophan depletion and kynurenine accumulation in the tumor microenvironment, thereby restoring T-cell activation and improving responses to immune checkpoint blockade (76). Specifically, we have expanded the discussion of tryptophan metabolism–targeted therapies by incorporating evidence from the phase III ECHO-301 clinical trial, which demonstrated that the IDO1 inhibitor epacadostat failed to improve progression-free survival when combined with pembrolizumab. Inhibitors of glutaminase, such as CB-839, suppress glutaminolysis that is upregulated in therapy-resistant melanomas, reducing proliferation of resistant cells and sensitizing them to targeted therapies (77). Enhanced glutamine targeting has been linked to improved antitumor immunity by reducing recruitment of suppressive myeloid populations and decreasing tumor kynurenine production, thus enhancing responsiveness to immune checkpoint inhibitors in preclinical models (77). Mechanistically informed combinations, such as glutaminase inhibition with PD-1/PD-L1 blockade or with MAPK pathway inhibitors, are being evaluated and underscore the importance of metabolic context in therapy design (78).

Beyond glucose and amino acid metabolism, lipid metabolic pathways have also emerged as regulators of tumor immunity. Increased FAO and lipid uptake in melanoma cells and tumor-associated immune cells can promote immune suppression and tumor persistence (79). Targeting FAO regulators such as CPT1A or inhibiting lipid uptake receptors such as CD36 has therefore been proposed as a strategy to restore antitumor immune responses (68). Emerging work further suggests modulating mitochondrial and oxidative metabolism may overcome resistance phenotypes that rely on OXPHOS and glutaminolysis. For example, in high-OXPHOS melanoma and brain metastasis models, glutaminase inhibition improved survival and supports the inclusion of metabolic agents in combination regimens for resistant disease (80). Beyond small molecules, metabolic reengineering of adoptive cell therapies (e.g., enhancing T cell mitochondrial fitness or glucose uptake) has potential to increase persistence and antitumor potency by making effector cells more resilient to metabolic stress *in vivo*.

Clinically, understanding and matching metabolic vulnerabilities—such as high LDH expression or glutamine dependency—may facilitate biomarker-driven patient stratification for metabolically informed combinations with immunotherapies, maximizing benefit while limiting off-target effects (81). Metabolism-targeted agents are now being tested with checkpoint inhibitors in early trials, underscoring a shift toward mechanism-guided combination strategies in melanoma immunotherapy.

## 4 Conclusion and outlook

Immune checkpoint blockade has transformed melanoma therapy, yet durable responses remain limited, reflecting deeply rooted resistance mechanisms beyond immune receptor signaling. Evidence reviewed here supports a unifying concept: metabolic reprogramming orchestrates tumor cell fitness, immune suppression, and therapy resistance. Melanoma cells shape a metabolically hostile microenvironment through altered nutrient availability, redox balance, and metabolite signaling, systematically constraining immune effector function and undermining checkpoint reinvigoration. Immune pressure itself selects for metabolically flexible tumor states—such as OXPHOS-high or lipid-rewired phenotypes—that evade elimination, highlighting a reciprocal interplay between metabolism and immune activity. Translationally, these insights argue for metabolism-guided therapeutic strategies, including biomarker-driven patient selection, rational combination therapies, and adaptive treatment sequencing. Emerging single-cell and spatial multi-omics approaches will enable precise mapping of metabolic heterogeneity and immune landscapes, providing actionable insights for intervention. Beyond melanoma, conserved metabolic mechanisms—amino acid depletion, lactate accumulation, lipid-driven immune dysfunction—inform immune resistance across solid tumors. Targeting tumor metabolism offers a path toward integrated, systems-level immunotherapies capable of durable antitumor responses.

## Glossary

ACAT2: acetyl-CoA acetyltransferase
ACT: adoptive T cell therapy
AHR: aryl hydrocarbon receptor
AMPK: AMP-activated protein kinase
ARG1: arginase 1
ASS1: argininosuccinate synthase 1
ATF3: activating transcription factor 3
CAF: cancer-associated fibroblast
CD3ζ: T-cell receptor zeta chain
CPT1A: carnitine palmitoyltransferase 1A
CREB: cAMP response element-binding protein
CTLA-4: cytotoxic T-lymphocyte–associated antigen 4
DC: dendritic cell
DUSP1: dual specificity phosphatase 1
EV: extracellular vesicle
FAO: fatty acid oxidation
FOXP3: forkhead box P3
GCL: glutamate-cysteine ligase
GCN2: general control nonderepressible 2
GLS: glutaminase
GLUT1: glucose transporter 1
GPX4: glutathione peroxidase 4
GSH: glutathione (reduced)
GSS: glutathione synthetase
GSSG: glutathione disulfide (oxidized)
GZMB: granzyme B
HIF-1α: hypoxia-inducible factor 1-alpha
HK2: hexokinase 2
I3A: indole-3-aldehyde
ICB: immune checkpoint blockade
ICI: immune checkpoint inhibitor
IDO1: indoleamine 2,3-dioxygenase 1
IFN-γ: interferon-gamma
iNOS/NOS2: inducible nitric oxide synthase
Kyn: Kynurenine
LAT1: L-type amino acid transporter 1
LDH: lactate dehydrogenase
LDHA: lactate dehydrogenase A
LPC: lysophosphatidylcholine
MAPK: mitogen-activated protein kinase
MCT: monocarboxylate transporter
MDSC: myeloid-derived suppressor cell
mTOR: mechanistic target of rapamycin
NFAT: nuclear factor of activated T-cells
NK cell: natural killer cell
NO: nitric oxide
OGDH: oxoglutarate dehydrogenase
OXPHOS: oxidative phosphorylation
PADI: peptidyl arginine deiminase
PD-1: programmed cell death protein 1
PD-L1: programmed death-ligand 1
PDHA1: pyruvate dehydrogenase E1 subunit alpha 1
PFK: phosphofructokinase
PFKFB3: 6-phosphofructo-2-kinase/fructose-2,6-biphosphatase 3
PKM2: pyruvate kinase M2
PPARγ: peroxisome proliferator-activated receptor gamma
PRMT: protein arginine methyltransferase
ROS: reactive oxygen species
SLC1A5: solute carrier family 1 member 5
SLC7A11: solute carrier family 7 member 11
SREBP: sterol regulatory element-binding protein
TAM: tumor-associated macrophage
TCA cycle: tricarboxylic acid cycle
TCR: T-cell receptor
TDO2: tryptophan 2,3-dioxygenase
TFEB: transcription factor EB
TIL: tumor-infiltrating lymphocyte
TME: tumor microenvironment
TNF-α: tumor necrosis factor-alpha
Trp: Tryptophan
Treg: regulatory T cell
VEGF: vascular endothelial growth factor
